# Ophthalmic Complications Following Sinus Lifting Surgery: Case Report and Review of Literature

**DOI:** 10.1002/ccr3.70902

**Published:** 2025-09-21

**Authors:** Nima Dehghani, Xaniar Mahmoudi, Mohadeseh Azarsina, Sahar Hassantash

**Affiliations:** ^1^ Department of Oral and Maxillofacial Surgery Tehran University of Medical Sciences School of Dentistry Tehran Tehran Iran; ^2^ Department of Oral and Maxillofacial Surgery Tabriz University of Medical Sciences Tabriz Iran; ^3^ Department of Estetic and Restorative Dentistry Tehran University of Medical Sciences School of Dentistry Tehran Tehran Iran; ^4^ Department of Public Health Sciences University of Miami Miller School of Medicine Florida USA

**Keywords:** complication, dental implant, maxillary sinus, sinus floor augmentation

## Abstract

The sinus lift procedure enables implant placement in the posterior maxillary region with insufficient bone volume. Our study presents a case of subconjunctival hemorrhage following an open sinus lift procedure. We discuss ophthalmic complications, including swelling and discoloration, and explore treatment methods for this rare yet significant complication.

## Graphical abstract

Pation showed subconjuctival hemorrhage sign, one day after sinus agumentation surgery.

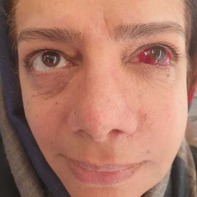

## 1 Introduction

The placement of implants in the posterior maxilla presents a significant challenge due to the limited bone volume and quality often caused by maxillary atrophy. To overcome this, sinus floor augmentation has been widely adopted, utilizing either the lateral window or osteotome techniques. Both methods are considered safe and effective, demonstrating high success rates for implants placed in grafted areas [1]. The primary goal of sinus lift surgery is to stimulate new bone formation in the floor of the maxillary sinus by utilizing the regenerative potential of the Schneiderian membrane, which lines the sinus cavity.

The proximity of the maxillary sinus to the orbital cavity introduces a risk of ophthalmic complications, which, although rare—occurring in approximately 0.01%–2.2% of cases—can be severe, potentially resulting in permanent dysfunction [2, 3].

Ophthalmic complications are categorized into three groups: minor (grade 1), which includes damage to the lamina papyracea; major (grade 2), involving damage to the lacrimal duct; and severe (grade 3), which encompasses retro‐orbital hemorrhage, optic nerve injury, any form of vision reduction or blindness, and damage to the ocular muscles [4, 5, 6]. The risk of such complications is influenced by several factors, such as the surgeon's experience, the patients' history of prior surgeries, the extent of the surgical procedure, and anatomical variations [7].

In addition to ophthalmic issues, the sinus lift procedure can lead to other potential complications, such as Schneiderian membrane perforation—the most common complication—postoperative infection, sinusitis, graft exposure, graft loss, swelling, bleeding, and emphysema [8, 9]. Recognizing and understanding these risks is essential for both planning the surgical procedure and informing the patient of potential outcomes.

Next, we will introduce the anatomy of the maxillary sinus and potential ophthalmic complications following sinus lift surgery.

### 1.1 Anatomy

The maxillary sinus, typically holding 12–15 mL of air in adults, has a pyramidal shape with its base adjacent to the nasal cavity. The upper part contributes to the orbital floor, while the apex extends toward the zygomatic bone. The sinus drains through the ostium, an opening in the upper inner wall, which functions as an overflow outlet. Anatomically, the maxillary sinus extends from the premolar or canine region anteriorly to the maxillary tuberosity posteriorly, with the floor usually reaching its lowest point around the first molar. In dentate individuals, the sinus floor aligns closely with the nasal floor, making it the thickest part of the walls. In contrast, in edentulous patients, it is generally about 1 cm below the nasal floor [10].

The blood supply to the sinus is provided by branches of the maxillary artery, including the posterior superior alveolar artery, inferior orbital artery, greater palatine artery, and sphenopalatine artery. During sinus lift procedures, injury to the posterior superior alveolar and inferior orbital arteries poses a risk of bleeding. Anatomical dissections have shown that the dental branch of the posterior superior alveolar artery may connect with the inferior orbital artery, though this is visible radiographically in only half of the cases. The close anatomical relationship between the maxillary sinus and orbit, along with these vascular connections, facilitates the spread of infections and other complications [11].

### 1.2 Possible Mechanisms Leading to Ophthalmic Complications During Sinus Lifting Surgery

Manipulation of the maxillary sinus during augmentation surgery can inadvertently affect the orbital floor or walls. If these bones are thin or compromised, they may fracture, damaging the muscles that control eye movement and potentially causing diplopia (double vision) [12, 13]. Injury to the ophthalmic artery or its branches may also lead to retrobulbar hemorrhage. The resulting hematoma increases intraorbital pressure (orbital compartment syndrome), which can compress the optic nerve, reduce blood supply, and risk vision loss [14].

Infections from the surgical site pose another serious threat, spreading directly into the orbit or via venous channels. Orbital cellulitis can result, leading to pain, inflammation, and potential vision impairment if not treated promptly [15]. Moreover, disruption of the normal pressure balance between the sinus and orbit may worsen pre‐existing conditions or create new ones. For example, creating an opening in the sinus can trigger pressure changes that affect surrounding tissues [16].

Understanding these mechanisms is vital for surgical planning and technique. Careful preoperative assessment, atraumatic handling, and immediate management of complications are essential to safeguard ocular health.

### 1.3 Ophthalmic Complications During Sinus Surgeries

#### 1.3.1 Orbital Hemorrhage

Orbital hemorrhage is a serious complication that can occur following sinus surgery, potentially leading to increased orbital pressure and compression of eye structures, which may ultimately result in visual impairment. The proposed etiologies for this condition include damage to the ophthalmic artery and its branches, as well as other vascular structures in the region. The use of anticoagulant and antiplatelet medications, which elevate the risk of bleeding, along with the presence of vascular anomalies within the orbit, also contribute to the susceptibility to injury. Patients presenting with orbital hemorrhage may exhibit symptoms such as proptosis, periorbital swelling and ecchymosis, pain, restricted eye movements, and visual disturbances [3, 7].

Management of orbital hemorrhage primarily focuses on reducing orbital pressure and controlling bleeding. Initial strategies include the application of cold packs, elevation of the patient's head, and administration of systemic corticosteroids to mitigate swelling. In severe cases where there is a risk to vision, surgical intervention may be necessary. In critical situations, procedures such as lateral canthotomy and cantholysis are carried out to alleviate orbital pressure in these critical situations [17, 18, 19]. Regular monitoring and ophthalmic evaluations are essential to assess the patient's vision and monitor for signs of compartment syndrome or other potential complications.

#### 1.3.2 Orbital Cellulitis and Abscess

Elevation of the sinus membrane during surgery can cause inflammation and swelling of the Schneiderian membrane. While this often resolves quickly, anatomical abnormalities in the ostiomeatal complex may delay healing and increase the risk of infection within the maxillary sinus [20]. Orbital cellulitis and abscess are serious complications arising from bacterial spread from the sinus to the orbit. Patients with compromised immunity, such as diabetes or immunodeficiency, are particularly vulnerable.

Immediate treatment is critical, and hospitalization is often required due to the risk of further spread and possible intracranial involvement. Management involves draining the infectious material, followed by broad‐spectrum antibiotics. Effective regimens may include clindamycin with third‐generation cephalosporins, vancomycin with or without meropenem, ampicillin–sulbactam, or cephalosporins combined with metronidazole. Nasal drops, such as saline and decongestants, should also be prescribed to improve drainage and reduce infection risk [21].

#### 1.3.3 Orbital Emphysema

Orbital emphysema is an uncommon condition that can occur following dental procedures where air is introduced into soft tissues, resulting in tissue swelling. The entry of air into these tissues typically leads to immediate swelling in the facial area, often presenting as crepitus upon palpation. This condition can also be accompanied by pain and functional difficulties due to localized compression [22]. The occurrence of emphysema is often linked to maneuvers performed either by the patient or the dental surgeon. Specific patient actions, such as sneezing with a closed mouth, blowing the nose, or playing wind instruments can introduce air into the soft tissue layers. Additionally, dental procedures involving the use of high‐speed handpieces and air/water syringes during extractions, periodontal therapy, orthognathic surgery, restorative and endodontic treatments, and maxillary sinus floor augmentation are also potential contributors to the development of emphysema [8, 23]. Studies indicate that iatrogenic causes are the most common reasons for emphysema [22]. Localized emphysema can affect malar and periorbital areas. While the association between periorbital emphysema and dental procedures in the posterior maxilla has been recognized, it is important to note that this condition can also arise spontaneously, without any direct manipulation [23, 24, 25, 26].

In cases with mild emphysema, monitoring the patient to manage potential complications is often sufficient. The application of a warm compress can expedite the healing process. Patients should also be cautioned against performing the Valsalva maneuver or other actions that might increase intranasal pressure. However, in severe cases with significant complications, more intensive interventions may be necessary [27, 28].

#### 1.3.4 Severe Ophthalmic Impairments (Globe Rupture, Blindness, etc.)

Instances of globe rupture following the penetration of zygomatic implants into the orbital cavity have been documented in the literature [29, 30]. Another serious but rare complication that can arise from maxillary sinus manipulation surgeries is significant visual impairment. Although these events are uncommon, their severe and often irreversible nature necessitates careful consideration [31].

The underlying mechanisms for such impairments include direct trauma to the optic nerve or globe, vascular damage such as injury to the posterior ciliary artery, and increased intraocular pressure, which can lead to optic nerve ischemia. The clinical symptoms associated with these complications range from acute or subacute vision loss, which may vary from mild blurring to complete blindness. Other possible symptoms include visual field defects, such as central or peripheral scotomas, abnormalities in color vision, the presence of a Marcus Gunn pupil, and optic disc edema or atrophy.

The treatment and management of these injuries depend on their severity. In cases where the visual impairment is minor and temporary, management typically involves patient observation and the administration of systemic corticosteroids. However, for more severe cases, surgical intervention to alleviate pressure around the optic nerve may be necessary. In every instance, consultation with an ophthalmologist is crucial [32, 33].

Early detection of visual issues, timely consultation with an ophthalmologist, and appropriate management are critical for achieving the best possible outcomes and preventing future complications.

## 2 Case History

**FIGURE 1:**
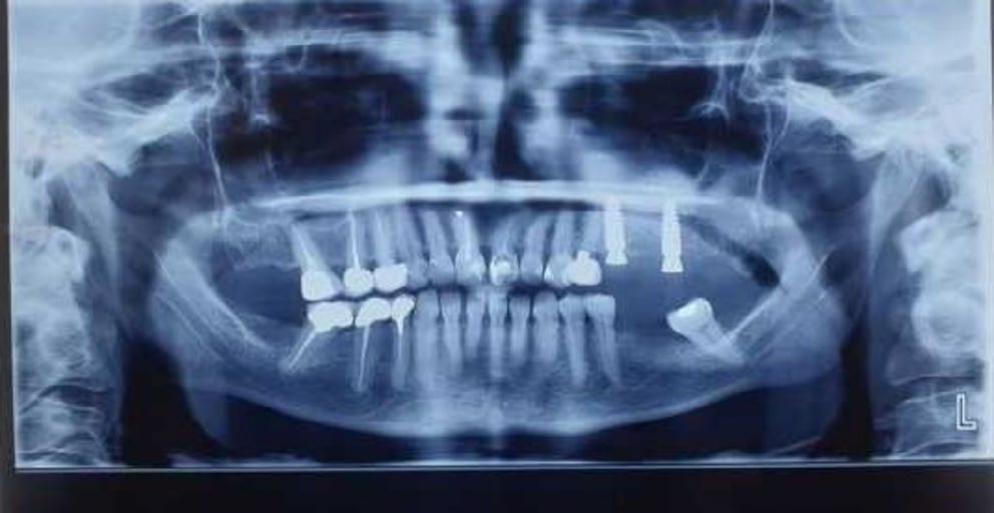
Panoramic radiograph showing sinus lifting and implant insertion in the right posterior maxilla. (Published with patient's consent).

A 53‐year‐old female patient with a history of controlled hypertension, without any recent blood pressure fluctuations, presented for the reconstruction of the upper right posterior maxillary area following the loss of teeth (Figure 1). During the same session, she underwent an open sinus lift surgery and the placement of two dental implants. The patient was discharged from the clinic without complications and was prescribed routine postoperative medications.

**FIGURE 2:**
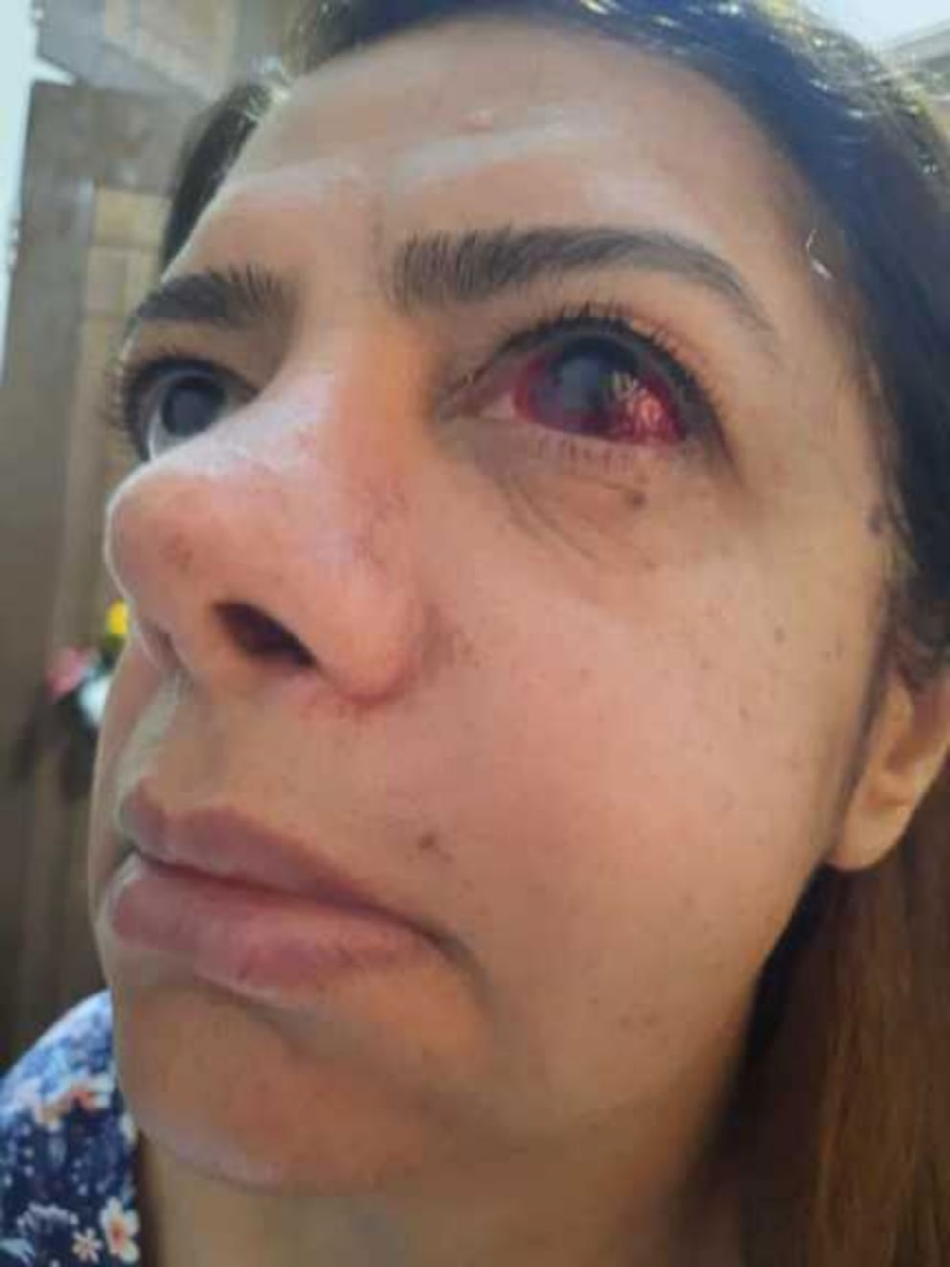
Patient's appearance on the day following surgery, highlighting subconjunctival hemorrhage in the right eye and noticeable swelling of the right cheek. (Published with patient's consent).

The following day, the patient returned with clinical signs of swelling around the right periorbital and malar areas, accompanied by subconjunctival hemorrhage (Figure 2). The swelling was mild, with no signs of infection, such as pus discharge or fever. The patient did not report any pain or ophthalmic complications like vision loss, blurred vision, or diplopia.

## 3 Investigations and Treatment

**FIGURE 3:**
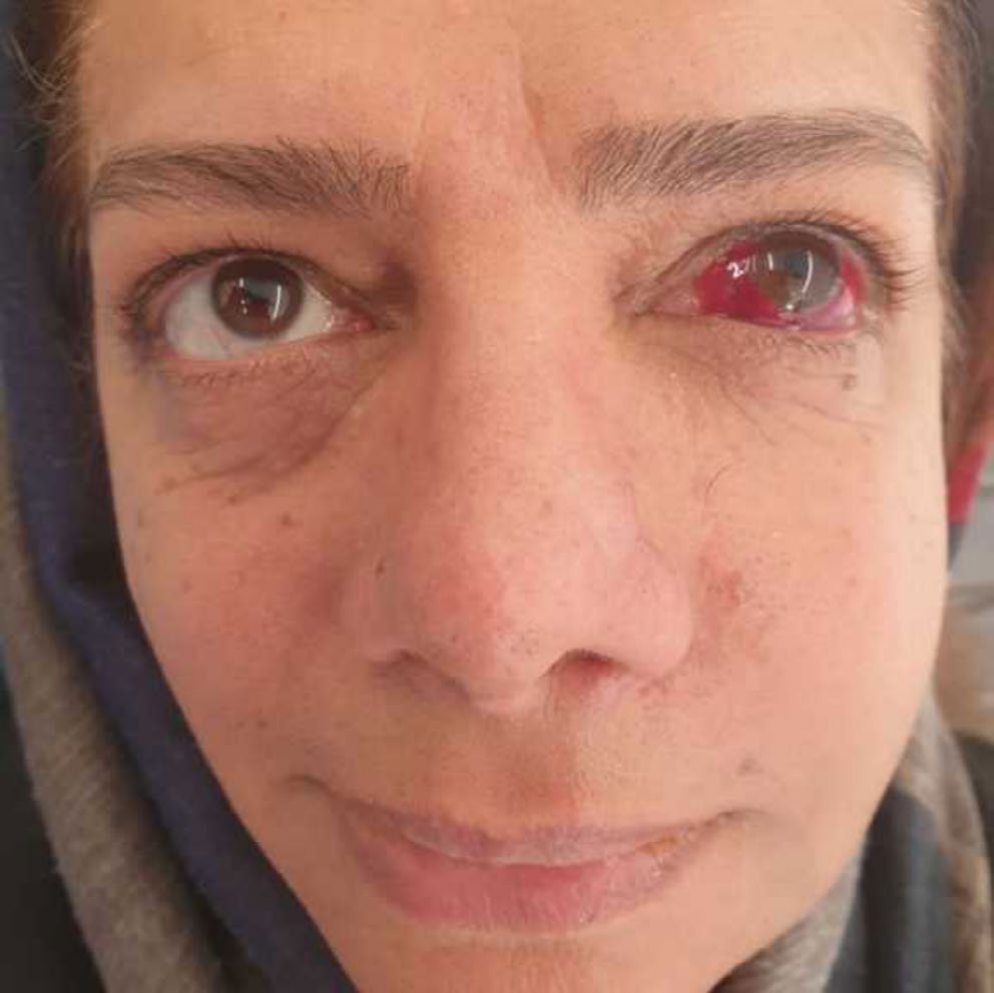
Postoperative view at 3 days. (Published with patient's consent).

The patient was promptly referred to an ophthalmologist for further evaluation. The ophthalmologist determined that antibiotic therapy was unnecessary and prescribed artificial tear eye drops and cold compresses. The patient was closely monitored to ensure that her symptoms did not progress (Figure 3). This complication is believed to have resulted from intraoperative or postoperative bleeding caused by manipulation and damage to blood vessels in the area during the sinus lift procedure. No intraoperative complications occurred during the sinus lift procedure. The Schneiderian membrane remained intact; there was no excessive bleeding, and the surgery was completed smoothly without any technical difficulties. Based on our assessment, the subconjunctival hemorrhage observed postoperatively was most likely due to trauma to the regional blood vessels during the procedure. Such trauma could have been caused during surgical flap elevation, the creation of the lateral window, dissection of the sinus membrane from the bony walls, or during crestal drilling for implant placement. Although no abnormal bleeding was observed intraoperatively, the patient's symptoms may have resulted from microtrauma or delayed bleeding, particularly considering the patient's history of hypertension.

A postoperative panoramic radiograph was taken, which showed proper graft positioning and implant placement. There was no evidence of sinus membrane perforation, bone graft displacement, or implant migration. Additionally, the patient's swelling and edema were subsiding, and there were no signs of progressive symptoms or uncontrolled bleeding. Therefore, in our clinical judgment, a CBCT scan was not indicated at that stage. The diagnosis was made based on clinical findings, and the patient was promptly referred to an ophthalmology center for further evaluation. A thorough ophthalmic examination was conducted, which revealed no pathological signs of any significant ocular injury. The absence of concerning findings was confirmed by the attending ophthalmologist.

To minimize the risk of similar complications, we emphasize the importance of performing the procedure as atraumatically as possible and taking great care to avoid unnecessary trauma to the surrounding tissues. Systemic factors were ruled out as the cause, given that both periorbital areas would likely have been affected otherwise. Despite its rarity, this complication can occur after sinus lift surgery, underscoring the need for clinicians to be fully aware of its management and patient care.

## 4 Outcome and Follow‐Up

**FIGURE 4:**
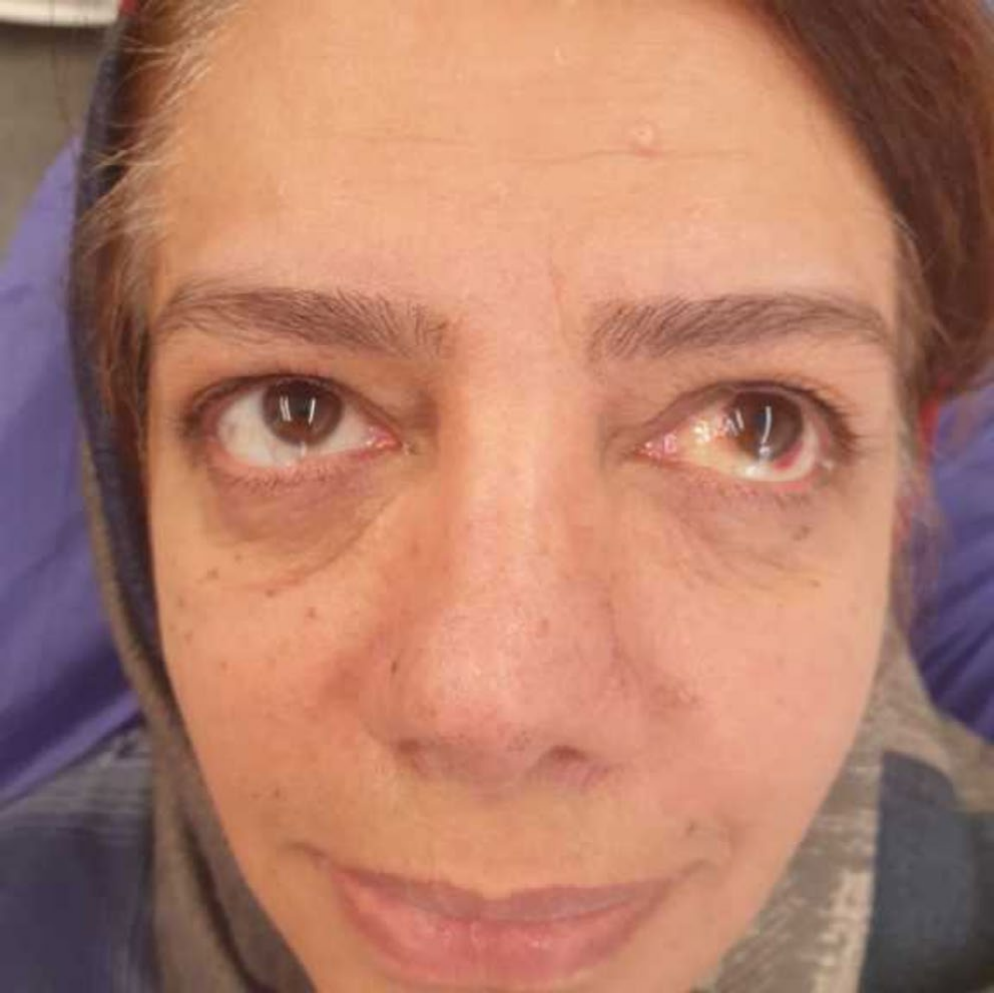
Postoperative view at 3 weeks, demonstrating healing process. (Published with patient's consent).

**FIGURE 5:**
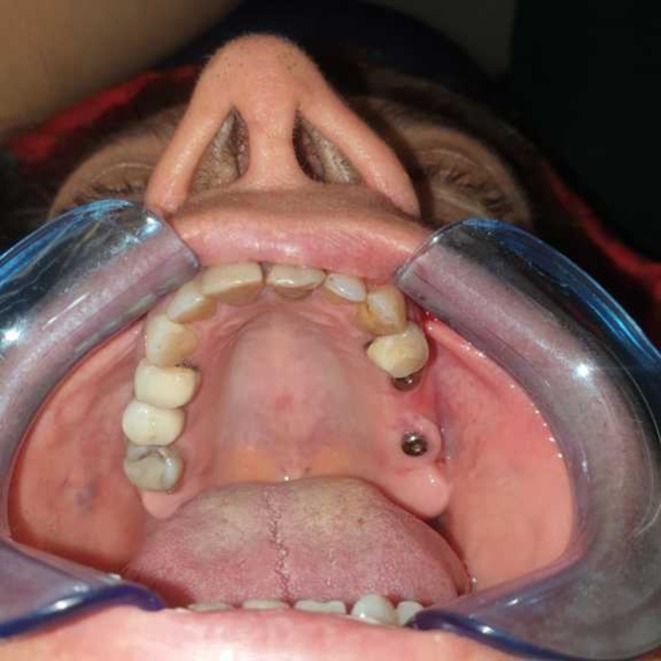
Occlusal view of the intraoral maxillary arch postimplant placement and sinus lift surgery, showing the alignment and integration of the implants within the arch after 3 weeks.

Two weeks later, the swelling and hemorrhage in the right periorbital area had significantly decreased. By 3 weeks postsurgery, the symptoms had completely resolved, and the patient was asymptomatic (Figures 4 and 5).

A comprehensive review of the literature revealed no prior reports of subconjunctival hemorrhage following sinus lift surgery. Therefore, while this complication is rare, it requires careful observation to ensure prompt relief of symptoms.

## 5 Discussion

In this article, we discuss a case who experienced subconjunctival hemorrhage following a sinus augmentation procedure. The incident occurred 1 day postsurgery, with the patient exhibiting no symptoms of visual impairment or blurred vision. Upon recognizing the condition, the patient was promptly referred to an ophthalmologist for further evaluation. The ophthalmologist determined that neither antibiotics nor corticosteroids were necessary. Over the following week, the swelling and subconjunctival hemorrhage significantly diminished, and by 3 weeks postsurgery, these symptoms had completely resolved. No complications arose related to the bone augmentation or implants in the affected area.

Periorbital emphysema and subconjunctival hemorrhage are recognized as rare clinical findings following maxillary sinus surgeries. While these conditions typically do not cause major issues or require intervention, there are cases where an increase in intraocular pressure and subsequent damage may necessitate surgical intervention to reduce the pressure. In such instances, it is crucial to differentiate between emphysema and hemorrhage, and infections or abscesses within the orbital space. In cases where infection is present, more aggressive interventions, including the administration of antibiotics, are required [34].

Subcutaneous emphysema is a potential complication that can arise following dental procedures and intraoral surgeries. The occurrence of this condition may be influenced by inappropriate patient maneuvers, the dentist's actions, and the instruments used during surgery [35]. While reports of similar incidents exist in the literature, they are relatively limited in number.

In a report from 2017, a patient developed periorbital emphysema and scleral erythema the day after undergoing sinus lift surgery. This was attributed to increased intranasal pressure caused by the patient. In this case, the patient did not experience any vision problems, and the condition resolved after 10 days of antibiotic and dexamethasone therapy [8]. Another case involved a patient who developed infraorbital emphysema just 2 h after an open sinus lift procedure. Suspecting a hemorrhage, the surgeon reopened the surgical site but found no specific bleeding point. The patient recovered and was discharged after 3 days of observation and intravenous antibiotic therapy [26].

A 2016 report described ocular complications following sinus lift surgery, where the patient experienced conjunctival edema 7 days postprocedure. A CT scan revealed mucosal thickening that entirely obstructed the maxillary sinus cavity, though the ostium remained patent. The patient reported epiphora but no vision problems or impairment. This condition resolved after 10 days of treatment with antibiotics and steroid eye drops [36].

In most cases of periorbital emphysema and conjunctival hemorrhage reported in the literature, patients have shown significant improvement over time without the need for specific interventions [8, 26, 36]. This was also observed in the present case. However, there are reports of emphysema spreading through fascial planes to distant areas such as the mediastinum and the parapharyngeal space, which can be life‐threatening. Therefore, early diagnosis and prompt intervention are crucial [37].

In the present case, the exact cause of subconjunctival hemorrhage is unclear. The patient reported no actions that would have increased intranasal pressure, and the Schneiderian membrane was intact. It is likely that the blood vessels in the area were damaged, and due to the proximity of the maxillary sinus to the eye globe, this condition occurred, despite no specific bleeding point being identified. In contrast, the case reviewed by Farina et al. [8], involved a patient who explicitly noted an increase in intranasal pressure leading to periorbital emphysema. In the case reviewed by Sakakibara et al. [26], emphysema developed after the patient blew the nose, while the case reviewed by Stacchi et al. was attributed to a probable infectious process [36]. To prevent potential emphysema after sinus lift surgery, it is essential to provide patients with clear and precise verbal and written instructions. In addition, surgeons frequently prescribe antibiotics to prevent infection and corticosteroids to reduce the inflammatory response [8, 26, 36].

Dental implant treatments sometimes require bone reconstruction, particularly in the posterior maxilla, where certain complications following surgical manipulation are inevitable [38]. However, various factors can contribute to these complications. According to the literature, the primary cause of complications like emphysema is often the patient's own actions, such as performing inappropriate maneuvers. Educating patients and providing clear verbal and written instructions can be effective in preventing such conditions [39].

Additionally, greater caution should be exercised concerning anatomical abnormalities that may increase the risk of complications, such as bleeding in the area. Surgery should be performed calmly, with care taken to avoid excessive trauma to the area. The use of piezosurgery devices to minimize trauma during surgery, along with modifications in flap design, can also help reduce the likelihood of complications following sinus lift procedures. In any case, prompt intervention and emergency consultation with an ophthalmologist are essential if any ophthalmic complications occur during or after surgery [40, 41, 42].

## 6 Conclusion

Despite the high success rate of sinus lift procedures, ophthalmic complications remain one of the most serious and potentially dangerous risks. It is crucial for all dentists and oral and maxillofacial surgeons who routinely perform sinus lift surgeries to be aware of the causes and management of these complications. Proper knowledge and prompt action can significantly reduce the risk and ensure better outcomes for patients.

## Author Contributions

**Nima Dehghani:** conceptualization, data curation, funding acquisition, investigation, methodology, project administration, resources, supervision, visualization, writing – original draft, writing – review and editing. **Xaniar Mahmoudi:** conceptualization, data curation, formal analysis, funding acquisition, investigation, project administration, resources, software, validation, visualization, writing – original draft, writing – review and editing. **Mohadeseh Azarsina:** conceptualization, data curation, formal analysis, funding acquisition, investigation, methodology, project administration, validation, writing – original draft, writing – review and editing. **Sahar Hassantash:** data curation, formal analysis, resources, software, validation.

## Ethics Statement

No ethics approval needed. Written informed consent has been obtained from the patient.

## Conflicts of Interest

The authors declare no conflicts of interest.

## Data Availability

The data that support the findings of this study are available upon request from the corresponding author.
